# The Anaerobic Power Assessment in CrossFit^®^ Athletes: An Agreement Study

**DOI:** 10.3390/ijerph18168878

**Published:** 2021-08-23

**Authors:** Tomás Ponce-García, Javier Benítez-Porres, Jerónimo Carmelo García-Romero, Alejandro Castillo-Domínguez, José Ramón Alvero-Cruz

**Affiliations:** 1 Department of Human Physiology, Histology, Pathological Anatomy and Sports Physical Education, University of Málaga, 29071 Málaga, Spain; benitez@uma.es (J.B.-P.); jeronimo@uma.es (J.C.G.-R.); 2 Department of Nursing and Podiatry, University of Málaga, 29071 Málaga, Spain; alejandrocastillo@uma.es

**Keywords:** anaerobic power, peak power, HIFT, high-intensity functional training, crossfit, athletes, field test

## Abstract

Anaerobic power and capacity are considered determinants of performance and are usually assessed in athletes as a part of their physical capacities’ evaluation along the season. For that purpose, many field tests have been created. The main objective of this study was to analyze the agreement between four field tests and a laboratory test. Nineteen CrossFit^®^ (CF) athletes were recruited for this study (28.63 ± 6.62 years) who had been practicing CF for at least one year. Tests performed were: (1) Anaerobic Squat Test at 60% of bodyweight (AST60); (2) Anaerobic Squat Test at 70% of bodyweight (AST70); (3) Repeated Jump Test (RJT); (4) Assault Bike Test (ABT); and (5) Wingate Anaerobic Test on a cycle ergometer (WG). All tests consisted of 30 s of max effort. The differences among methods were tested using a repeated-measures analysis of variance (ANOVA) and effect size. Agreement between methods was performed using Bland–Altman analysis. Analysis of agreement showed systematic bias in all field test PP values, which varied between −110.05 (AST60_PP_—WG_PP_) and 463.58 (ABT_PP_—WG_PP_), and a significant proportional error in ABT_PP_ by rank correlation (*p* < 0.001). Repeated-measures ANOVA showed significant differences among PP values (*F*(1.76,31.59) = 130.61, *p* =< 0.001). In conclusion, since to our knowledge, this is the first study to analyze the agreement between various methods to estimate anaerobic power in CF athletes. Apart from ABT, all tests showed good agreement and can be used interchangeably in CF athletes. Our results suggest that AST and RJT are good alternatives for measuring the anaerobic power in CF athletes when access to a laboratory is not possible.

## 1. Introduction

Anaerobic capacity has been defined as the total amount of ATP re-synthesized, by the whole body, during a maximal intensity and short duration effort by means of the anaerobic metabolic pathways [1]. The time interval to best measure the anaerobic capacity is 30 s [2] since up to 80% of the energy consumed in 30 s of maximal effort comes from anaerobic sources [3,4]. In addition, in a longer test, individuals tend not to apply the maximum intensity [5]. There are several laboratory tests to assess the anaerobic performance [6]. However, most are expensive and difficult to perform due to the specific equipment they require. For that reason, one of the most widely used laboratory tests to assess this ability is the Wingate test, which consists of pedaling with arms or legs at maximum effort for 30 s against a resistance determined by the participant’s body weight. WG has shown to be a reliable test, having a test-retest correlation in many populations ranging from 0.89 to 0.98 [7]. Two main variables are determined from this test, peak power (PP) and mean power (XP). PP is also known as “anaerobic power” and is determined by the peak mechanical power recorded during the test, normally occurring in the first 5 to 10 s. In addition, XP is considered by many authors as the “anaerobic capacity” and represents the average mechanical power maintained during the 30 s, taken at 1, 3 or 5 s periods [7]. Some authors have shown PP and XP to be associate with performance in some team and individual sports, especially those performed at high intensity or a combination of low-moderate intensities with higher intensity peaks such as CF [8], surfing [9], alpine ski [10], soccer [11], track and field athletes [12] and many others.

In order to assess this ability out of the laboratory, numerous field tests, consisting of different exercises or tasks, have been created. Some of them based on different modalities of jumps [5,12,13,14,15,16]; running [14,17,18]; squat exercise [14,19,20]; and other exercises such as skipping [21]. All those tests have been studied in active individuals [17,18,21,22] as well as athletes of different sports such as soccer [14], volleyball [5,15], track and field [7,12,20,23], and cyclists [24,25]. They have shown to be valid tools to assess these parameters in athletes [5,12,18,19].

In the last decade, Functional Fitness Training has become one of the top fitness trends around the world [26,27]. One of these functional fitness programs, which has developed into a competitive sport, was branded as CrossFit^®^. CF is a multimodal high-intensity functional training program that combines weightlifting, gymnastics and athletics, among other movements in just one training or competition bout and develops all physical domains such as endurance, strength, stamina, etc. [28]. The multimodality characteristic of this sport, combined with the fact that the tests carried out in competition are not previously announced or standardized, means that CF athletes must be prepared for the unknown and therefore have an optimal development of all physical capacities such as maximum strength, stamina, power, speed, cardiorespiratory fitness, etc. [8,29,30,31,32,33,34,35]. Additionally, its intensity component indicates that CF competitors must exhibit a great deal of anaerobic performance to excel in this sport [29].

When a field test is developed to assess any ability of the athletes throughout the season, experts attempt to simulate the specific sporting gestures of the discipline for which it is created (running in soccer, for example). In the case of CF, as a multimodal sport made up of many elements of different kinds (squatting, jumping, running, lifting, etc.), it might seem challenging to succeed in choosing a specific exercise that encompasses all the skills and abilities necessary for this activity and evaluate any capacity accurately. Nevertheless, taking into account the specific characteristics of these athletes, it may be assumed that any field test might be a valid and interchangeable tool to assess any of the physical capacities. Hence, they might show a good performance in any test with jumping, running, cycling, squatting, etc.

In the current work, to assess the anaerobic performance by different exercises and determine their validity and level of agreement, four tests were chosen: a continuous jump test used in previous work by Dal Pupo et al. [5] (RJT), as well as three other tests that, to our knowledge, have not been used previously: two weighted deep squat tests (AST60 and AST70) at different percentages of the athlete’s bodyweight (60% and 70%) and a test performed with a particular machine used in CF where upper and lower limbs are used simultaneously called Assault Bike^®^ (ABT).

In CF athletes, some authors have evaluated the physiological determinants of performance in [8,30,31,32,33,34,35]. Most of them using laboratory tests to assess both the aerobic or anaerobic capacities and comparing the results with those obtained in standardized CF workouts. However, no study of agreement between field methods has been found. Therefore, the main purpose of this study is to analyze the agreement between four different modalities of field test measuring anaerobic performance (AST60, AST70, RJT and ABT) against the gold standard, Wingate test, in CF athletes.

## 2. Materials and Methods

### 2.1. Participants

Nineteen CF participants volunteered to participate in this study, approved by Málaga University Ethics Committee (CEUMA: 43-2018-H). They were experienced athletes who followed the same competitors’ training program and had competed in some national or international competition. Data collection was carried out over four weeks off-season. Except for the rest periods established before each test, the athletes followed their regular training regimen throughout those weeks. They were asked to stop taking any supplementation or performance-enhancing products one week prior to data collection. The participants were recruited and tested in a local CF center. All participants provided written informed consent. As inclusion criteria, a minimum of one year of CF practice was established. Any participants with the presence or suspicion of any cardiac pathology, suffering or having suffered recently any musculoskeletal injury or any other condition that prevented exercising properly were excluded. Descriptive data are shown in Table 1.

### 2.2. Study Design

**Figure 1:**
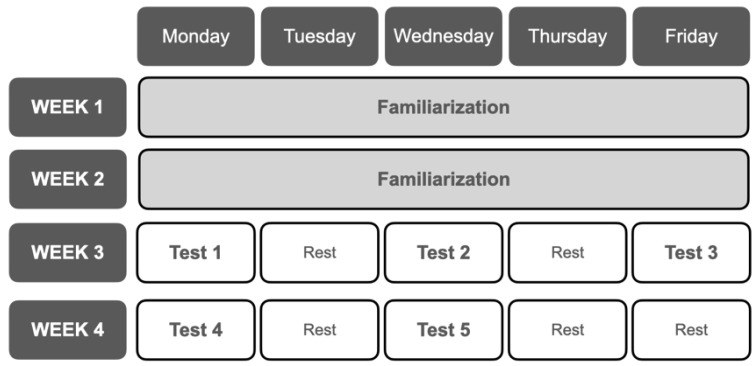
The chronology of the tests.

A cross-sectional study was conducted over four weeks. Despite the fact that all participants were familiar with the exercises in all tests, a familiarization session was also scheduled during the first two weeks. All trials were separated by at least 48 h and performed at the same daytime to avoid the effects of circadian rhythms [36]. Participants were also advised to refrain from any strenuous physical activity in the previous 24 h of each trial. Tests performed were: (1) Anaerobic Squat Test at 60% of bodyweight (AST60); (2) Anaerobic Squat Test at 70% of bodyweight (AST70); (3) Repeated Jump Test (RJT); (4) Assault Bike Test (ABT); and (5) Wingate Anaerobic Test on a cycle ergometer (WG). Tests order execution was randomly assigned. The chronology of the tests is shown in Figure 1.

### 2.3. Procedures

#### 2.3.1. Anthropometry, Body Composition and Other Physiological Variables

On the first day, to detect any possible cardiac pathology, all participants underwent an electrocardiogram assessed by a qualified physician. Furthermore, some anthropometric data were taken; height, by a wall-mounted stadiometer (SECA^®^ 206; SECA, Hamburg, Germany) with a precision of 1 mm and body mass, by a scale with a precision of 100 gr (SECA^®^ 803; SECA, Hamburg, Germany). Additionally, body composition was measured by a Medisystem Multifrequency Impedanciometer (Sanocare Human System SL, Madrid, Spain). Participants were asked to go fasting or without consuming any drink or food for at least 4 h, not having consumed alcohol in the last 48 h nor diuretics in the last 7 days or having performed strenuous physical activity in the previous 12 h [37]. Before the measure, they remained supine for 5 min with the upper limbs positioned about 30 degrees apart from the trunk and the lower limbs about 45 degrees apart [38]. Fat mass in kg was estimated according to Segal’s formula [39], Lean body mass in kg was calculated by subtracting fat mass from total body mass and muscle mass in kg according to Janssen’s formula [40]. Body composition variables were also calculated as a percentage (Table 1).

#### 2.3.2. All-Out Anaerobic Tests

##### Anaerobic Squat Test (AST60 and AST70)

**Figure 2:**
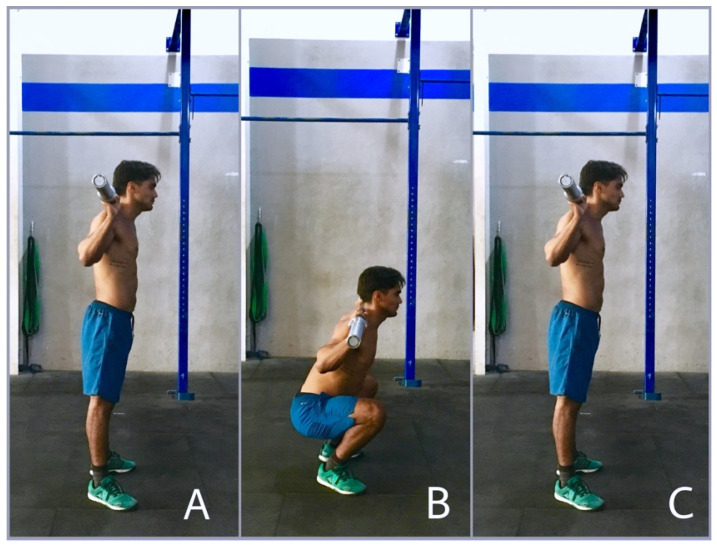
Full squat movement requirements. (**A**): start position; (**B**): lowest position; (**C**): final position.

The AST consisted of 30 s at the maximum effort of deep squats with a percentage of the participant bodyweight. The maximum number of squats had to be performed within that interval. Deep squat was established as a squat in which the iliac crest is below the highest part of the knee in its lowest position, and the leg, thigh and trunk segments are fully aligned at the highest position (Figure 2).

**Figure 3:**
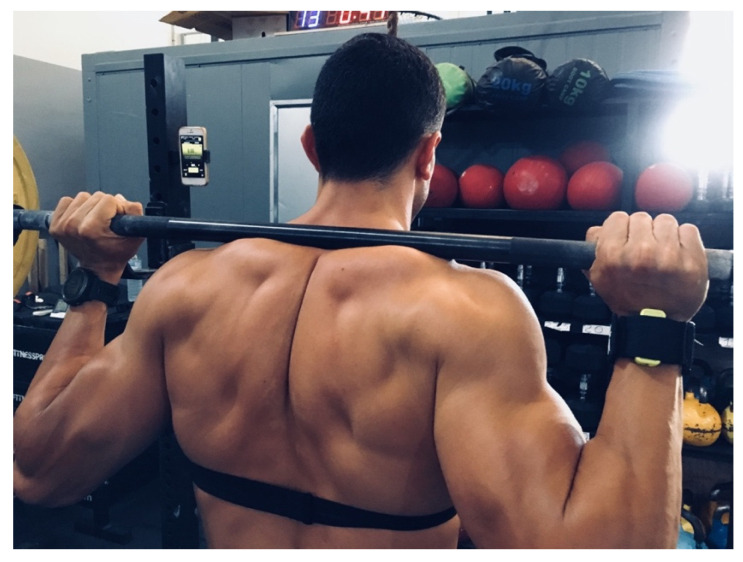
Beast sensor placement on the athlete’s at right wrist.

The equipment used was a standard olympic lifting set composed of a 20 kg barbell, plates between 5 and 15 kg, with increases of 5 kg, and fractional discs from 0.5 and 2.5 kg, with 0.5 kg increments, from Xenios Usa^®^ (Xenios Usa LLC, New York, NY, USA). The power of each repetition was registered by Beast^®^ accelerometry sensor (Beast technologies) attached to the participant’s wrist through a bracelet “ad hoc” (see Figure 3) and data processed by its smartphone application. Beast^®^ sensor has shown to be a valid and reliable tool to measure full-squat values [41]. Two trials with different loads were executed, 60% (AST60) and 70% (AST70) of participant bodyweight. Participants were weighed before each trial to determine the barbell load, rounded to the closest 0.5 kg. As a warm-up, they started with five minutes easy run, followed by one set of ten repetitions with an empty barbell, two more sets of ten repetitions with the assigned percentage and finished with 5 min easy run. Afterwards, a 5 min interval for recovery was established and used to set the accelerometry sensor. At the count of 3, 2, 1… “Go!” the participant began to work at maximum effort, trying to execute as many squats as possible, being verbally motivated by the examiner throughout the test. To cool down, they were asked to easy walk for 5 min.

Peak power (PP), mean power (XP) and minimal power (MP) were determined. Fatigue index (FI), understood as the loss of power during the 30 s interval, was calculated by the following formula FI (%) = (PP − PM/PP) × 100 [7].

##### Repeated Jump Test (RJT)

As previously described by Dal Pupo et al. [5], this test consisted of the maximum number of countermovement jumps in 30 s at the maximum height. Before the trial, participants warmed up with 5 min easy run, 3 sets of 10 forward jumps, 3 sets of 5 vertical jumps and 5 additional minutes easy run. Afterwards, a 5 min interval was established to rest and set the sensors. At the count of 3, 2, 1… “Go!” the participant started to jump as high and fast as possible. In order to keep the maximum intensity, the participant was encouraged by the researchers during the whole interval. Right after the test, they were asked to easy walk for 5 min to calm down. Jumping variables were registered by a Polar^®^ V800 with Running Bluetooth^®^ Smart. This sensor has been shown to be valid and reliable to determine jumping variables [42]. PP, XP, MP and FI were determined.

##### Assault Bike Test (ABT)

**Figure 4:**
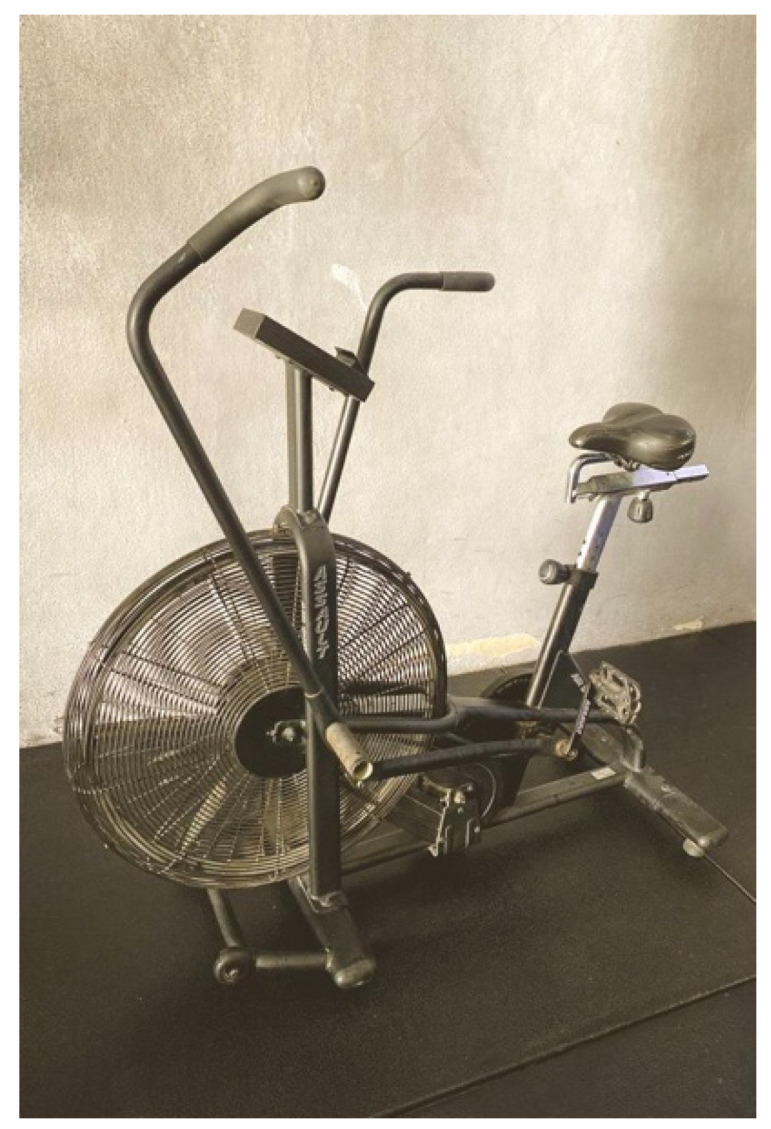
Assault Bike^®^ Classic.

This test was performed with an Assault Bike^®^ Classic model (Assault Fitness Products; Carlsbad, CA, USA). The Assault Bike^®^ is an air-resisted bike with the peculiarity of using both upper and lower extremities simultaneously (Figure 4). This machine has gained its popularity by being used by most CF centers and official competitions worldwide. The test consisted of 30 s at maximal effort. It began with a 15 min warm-up of cycling at 50 rpm (approximately 176 watts). Next, a 5 min recovery interval was established. The test was carried out from a static position without any inertia. To facilitate the initial start, the crank of the dominant leg was previously set to 45 degrees.

At the count of 3, 2, 1… “Go!” the participant started to ride as fast as possible. In order to keep the maximum intensity, the researcher motivated them verbally during the whole interval. A 5 min recovery interval ride at a warm-up pace was set to calm down. Power values were registered every 5 s. PP, obtained by PP(W) = maximal power output in the first 10 s; XP calculated by XP(W) = ∑ of each 5 s Power(W)/6; MP, as MP(W) = minimal power output in the last 10 s, and FI were determined.

##### Wingate Anaerobic Test (WG)

The wingate anaerobic test is considered the gold standard when measuring the anaerobic capacity and consists of 30 s at maximum speed on a cycle ergometer with a constant resistance of 0.075 kp per kg bodyweight [7]. The test was executed with a Monark 828E cycle ergometer (Monark Exercise AB, Vansbro, Sweden) calibrated before each trial. Since trials were completed in morning-time (between 9:00 am and 2:00 pm), the warm-up was extended from 5 to 15 min, as proposed by Souissi et al. [36]. To warm up, all participants were asked to ride at 50–70 rpm at 1 kp (50–70 watts) for 15 min. Afterwards, they took a 5 min recovery interval. Straightaway, at the count of “3, 2, 1… Go!” the participant started to ride as fast as possible. The researcher motivated them verbally during the whole time. A 5 min recovery ride at a warm-up pace was set to calm down. Every 5 s, power values were registered. PP, XP, MP and FI were determined.

### 2.4. Statistical Analysis

The Statistical Package for the Social Sciences (SPSS 21, IBM Corp., Armonk, NY, USA) and MedCalc Statistical Software (MedCalc 18.6, MedCalc Software Ltd., Ostend, Belgium) were used to carry out statistical analyses. The level of significance was set at *p* ≤ 0.05. Data were checked for normality by the Shapiro–Wilks analysis, and the agreement for the PP of the four methods was performed by using Bland–Altman analysis [43]. In order to evaluate the proportional error, Tau Kendall’s rank correlation of the difference and mean of every method paired with WG was carried out. Previously, variables of difference and mean were computed for each pair. Furthermore, the differences among PP, XP, MP and IF of the five methods were tested for statistical significance (*p* < 0.05) using a repeated-measures analysis of variance (ANOVA). When a significant difference was found, post hoc 2-tailed paired *t*-tests to determine which values were significantly different were used. The Bonferroni adjustment was applied to keep the overall significance level at 0.05. The assumption of sphericity was tested using Mauchly’s test. Additionally, the pairwise effect size was calculated by Cohen’s d using G*Power 3.1.9.6 software.

## 3. Results

All variables showed a normal distribution in Shapiro–Wilks analysis (*p* => 0.05), except for RJT_XP_ (*p* = 0.001) and RJT_MP_ (*p* = 0.022). Since the sphericity was violated, Greenhouse–Geisser corrected results are reported (ε = 0.44). The repeated-measures ANOVA showed significant differences among PP values, (*F*(1.76,31.59) = 130.61, *p* =< 0.001). Pairwise effect sizes are shown in Table 2. Additionally, absolute PP, XP and MP values of the AST60 and AST70 tests were slightly lower than the reference test. AST60 PP, XP and MP underestimated WG values by −110.05 (−14.12%), −101.07 (−15.20%) and −94.11 (−17.37%) watts, respectively. AST70 also underestimated WG values by −75.11 (−9.64%), −68.38 (−10.29%) and −56.16 (−10.37%) watts. In addition to the minor underestimation, the differences between AST70 and WG remained quite regular among all power values, around 10%, which was the only test that showed not statically significant differences by ANOVA test and showed the smallest effect size in all variables (Table 2).

In contrast, the homologous absolute values RJT and ABT were notably higher. With an overestimation of RJT values of 343.22 (44.06%), 393.24 (59.16%) and 380.21 (70.18%), and ABT values of 463.58 (59.52%), 286.05 (43.04%) and 262.10 (48.38%) (Table 2).

In addition, Bland–Altman’s analysis of agreement showed systematic bias in all field test PP values (*p* > 0.05). The smallest difference between all PP values and WG_PP_ was observed for the AST70 with an underestimation of −75.11 watts (95% CI, −124.80, −25.41). AST60 also underestimated PP by −110.05 watts (95% CI, −157.74, −62.36). Nevertheless, the other two tests, RJT and ABT, overestimated PP by 343.22 watts (95% CI, 312.63, 373.80) and 463.58 watts (95% CI, 380.18, 546.98), respectively.

**Figure 5:**
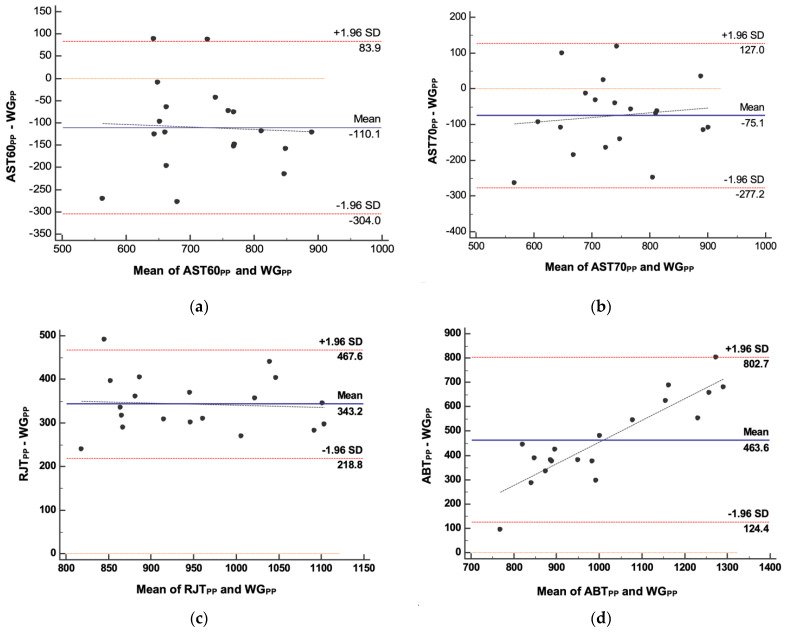
Bland–Altman’s plots representing differences (Y axes) and mean (X axes) of measurements between: (**a**) AST60 and WG; (**b**) AST70 and WG; (**c**) RJT and WG; (**d**) ABT and WG.

Furthermore, only a significant proportional error was found in ABT_PP_ by Tau Kendall’s rank correlation (Table 3 and Figure 5d).

## 4. Discussion

The main purpose of the present study was to evaluate the agreement between the five methods to assess anaerobic power in CF athletes. Since to our knowledge, this is the first study to analyze the agreement between various methods to estimate anaerobic power in CF athletes. Bland–Altman’s analysis revealed a systematic bias with a mean difference that can vary between −110.05 watts (AST60_PP_−WG_PP_) and 463.58 Watts (ABT_PP_−WG_PP_). Despite the systematic bias shown by all the field tests compared with the laboratory test, the results showed good agreement between all methods (*p* > 0.05) since more than 80% of the dots on the graph were within the limits of agreement. In contrast, Tau Kendall’s rank correlation analysis showed a proportional error in ABT_PP_ (*p* < 0.001), where the differences were small for low PP values in the range of measurements and become higher as the true value increases. Additionally, the lowest within-subject variability in all the variables studied in the present work suggests that the AST70 is a valid field test to assess the power and anaerobic capacity in CF athletes.

Some of the field tests practiced in this study, such as AST and ABT, have not been previously used. AST is a test based on the squat exercise tested with two different percentages of the participants’ body mass (60 and 70). The underestimation of PP absolute values, supported by the findings of Luebbers et al. [20], suggests that it might be interesting to replicate the study using higher percentages (75 and 80) to achieve more accurate agreement. In addition, some studies have shown underestimation of absolute values in a running test assessed in armed forces operators [21] and cycling athletes [25], as well as a kicking test studied in taekwondo athletes [44]. On the other hand, the overestimation of the RJT PP value is consistent with the findings of Sands et al. [16], where absolute power values of the Bosco test were higher than WG. In our study, overestimation was also found in ABT, and it might be due to the simultaneous use of lower and upper limbs to generate power instead of only the lower limbs as in WG. We have not found any previous study carried out with this machine that can provide data in this regard. However, the simultaneous use of the muscles of the lower limbs involved in pedaling and those of the upper limbs involved in pulling and pushing may suggest a more significant muscle mass implication and thus a greater capacity to generate power.

The results abovementioned are consistent with the WG_PP_ differences reported by Gacesa et al. [45] in a comparison testing of maximum anaerobic performance on different elite athletes. Their findings suggest that the ability to generate power may be dependent on the activity since the highest values were found in anaerobic predominant sports such as volleyball, basketball, hockey, boxing, and wrestling, and lower values in soccer, rowing, and long-distance running athletes, which are predominantly aerobic types of sports. Further, some authors have found differences in power values between participants of different positions in basketball [46] and elite runners of different distances [23]. Consequently, it might be thought anaerobic power to be related to specific disciplines or attributed to some degree of specificity of the athletes tested. However, the results shown in the present study, due to the need of CF athletes to face multiple physical demands with a high level of intensity, may indicate that these athletes are able to exhibit outstanding anaerobic performance in tests of different nature (jumping, squatting, cycling, etc.).

Many comparisons or validity studies where authors studied the level of agreement between only one field test and WG were found. However, a lack of agreement works between more than one field method and the laboratory test in the literature makes it difficult to compare our results with any other. Moreover, as mentioned above, most of their results show some level of under or overestimation of field-test values which may be attributable to the biomechanical, technical or any other difference in the sporting gesture used for each test together with the intrinsic characteristic of the athlete tested. Future studies analyzing the agreement between different task tests may be of interest to find the cause of that variability and the most suitable field test for each discipline, especially in a multimodal sport as it is CF.

One limitation of the present work was not considering any other variables, such as kinematics, that could reflect the different biomechanical or lifting strategies related to performance in AST or any other test. Future research should aim to record these variables mentioned above and evaluate the interaction in the outcomes, replicating this work with other tests composed by other CF-specific exercises or in athletes of different experience/fitness levels.

In practice, the use of AST70 or RJT as a method to assess the anaerobic power in CF athletes could provide an alternative for coaches interested in assessing or monitoring their athletes at any point of the season without the need of taking them to a sports medicine laboratory.

## 5. Conclusions

Since to our knowledge, this is the first study to analyze the agreement between various methods to estimate anaerobic power in CF athletes. In conclusion, our results show a good level of agreement between all four methods and WG, being greater in AST70, which suggests that they may be used interchangeably with the exception of ABT. The proportional error found in ABT might make its use doubtful. Moreover, the results of the present study suggest that the magnitude of peak power values seems to be dependent on the type of exercise and athlete characteristics.

## Figures and Tables

**Table 1:** Descriptive data of the sample (*n* = 19).

	Mean	SD
Age (years)	28.63	6.62
Height (cm)	176.18	5.34
Body Mass (kg)	81.67	6.43
Body Mass Index (kg/m^2^)	26.29	1.34
Fat Mass (kg)	24.71	6.35
Fat Mass (%)	20.10	5.18
Muscle Mass (kg)	35.03	3.74
Muscle Mass (%)	42.87	2.69
Lean Body Mass (kg)	56.95	10.02
Lean Body Mass (%)	79.90	5.18

**Table 2:** Absolute values of peak, mean, minimal power and fatigue index of the tests.

	PP			XP			MP			FI		
	Mean (±SD)	*p*	*d*	Mean (±SD)	*p*	*d*	Mean (±SD)	*p*	*d*	Mean (±SD)	*p*	*d*
AST60	668.84 (±98.05)	0.001	1.11	563.59 (±91.06)	0.001	1.20	447.63 (±98.64)	0.007	0.94	33.47 (±8.78)	1.0	0.30
AST70	703.79 (±112.94)	0.052	0.73	596.28 (±121.61)	0.182	0.60	485.58 (±111.84)	0.627	0.46	31.43 (±10.03)	1.0	0.13
RJT	1122.11 (±97.70)	<0.001	5.40	1057.90 (±154.65)	<0.001	3.56	921.95 (±113.29)	<0.001	4.69	17.79 (±7.25)	<0.001	1.39
ABT	1242.47 (±249.82)	<0.001	2.68	950.71 (±151.36)	<0.001	2.82	803.84 (±89.51)	<0.001	3.99	33.73 (±9.98)	0.570	0.47
WG	778.89 (±102.30)			664.66 (±73.08)			541.74 (±50.42)			29.71 (±8.39)		

AST60, anaerobic squat test at 60% of body weight; AST70, anaerobic squat test at 70% of body weight; RJT, repeated jump test, ABT, assault bike test; PP, peak power; XP, mean power; MP, minimal power; FI, fatigue index; SD, standard deviation; *p*, ANOVA *p*-values; *d*, pairwise effect sizes.

**Table 3:** Agreement analysis results.

Methods		Bias		Limits of Agreement				Kendall’s Tau	Absolute Percentage Error	
	Diff	95% CI	*p*	Lower	95% CI	Upper	95% CI	*p*	Median	95% CI
AST60—WG	−110.05	−157.74 to −62.36	0.0001	−303.98	−386.95 to −221.00	83.87	0.90 to 166.85	0.25	14.86%	12.00 to 17.77
AST70—WG	−75.11	−124.80 to −25.41	0.0052	−277.19	−363.65 to −190.72	126.98	40.51 to 213.44	0.89	12.20%	6.87 to 17.22
RJT—WG	343.22	312.63 to 373.80	<0.0001	218.84	165.62 to 272.06	467.59	414.38 to 520.81	0.58	42.19%	37.65 to 49.16
ABT—WG	463.58	380.18 to 546.98	<0.0001	124.44	−20.67 to 269.55	802.72	657.61 to 947.83	<0.001	59.48%	49.41 to 70.87

AST60, anaerobic squat test at 60% of body weight; AST70, anaerobic squat test at 70% of body weight; RJT, repeated jump test, ABT, assault bike test; CI, confidence Interval.

## Data Availability

The datasets used and/or analyzed during the current study are available from the corresponding author on reasonable request.
